# Supplementary material for: From sequence to enzyme mechanism using multi-label machine learning
Source: BMC Bioinformatics. 2014 May 19;15:150. doi: 10.1186/1471-2105-15-150 (PMC4229970; doi:10.1186/1471-2105-15-150)
Supplement: Additional file 2 — Java code of ml2db. Additional file ml2db_code.tar.gz contains the Java source code to run the multi-label machine learning experiments and save the results to database. The code’s Javadoc is included. [file 1471-2105-15-150-S2.zip › additional file 2/ml2db/utils/doc/index-files/index-6.html]

F-Index


JavaScript is disabled on your browser.


- Overview
- Package
- Class
- Use
- Tree
- Deprecated
- Index
- Help

- Prev Letter
- Next Letter

- Frames
- No Frames

- All Classes

A B C D E F G H I J K L M N O P Q R S T U V W X Y 


## F

fileChooserWithPreMessage(String, String) - Static method in class uk.ac.standrews.utils.main.guiutils.GuiUtils
:   1.

fileNotWritablePopUp(String) - Static method in class uk.ac.standrews.utils.main.FileUtils
:   Error Pop-up: file not writable

fileSize(String) - Static method in class uk.ac.standrews.utils.main.FileUtils
:   Get a file size (length in bytes)

fileToArrayOfArrays(String, String, boolean) - Static method in class uk.ac.standrews.utils.main.FileUtils
:   Reads a special-character-separated file (eg comma or tab separated) and
    creates an array of rows-columns.

fileToArrayOfLines(File) - Static method in class uk.ac.standrews.utils.main.FileUtils
:   Reads a file and adds the lines to an array, adapted from:
    http://www.computing.net/programming/wwwboard/forum/12276.html

fileToArrayOfLines(String) - Static method in class uk.ac.standrews.utils.main.FileUtils
:   Reads a file and adds the lines to an array, adapted from:
    http://www.computing.net/programming/wwwboard/forum/12276.html

fileTokensToArray(String, String) - Static method in class uk.ac.standrews.utils.main.FileUtils
:   Reads a file and adds the tokens (identified by regular expressions) to
    an array (e.g.

FileUtils - Class in uk.ac.standrews.utils.main
:   Utils to read, write files etc.

FileUtils() - Constructor for class uk.ac.standrews.utils.main.FileUtils


FileUtilsTest - Class in uk.ac.standrews.utils.test
:   Class

FileUtilsTest() - Constructor for class uk.ac.standrews.utils.test.FileUtilsTest


flipMap(TreeMap) - Static method in class uk.ac.standrews.utils.main.maputils.MapUtils
:   Substitutes keys and values in a map.

formatPercentOneFraction(double) - Static method in class uk.ac.standrews.utils.main.NumberUtils
:   Takes a double with any number of decimals and returns a String
    representation of a percentage 0.85842 -> 85.8%

from1DigitTo2DigitsDate(String) - Static method in class uk.ac.standrews.utils.main.TimeUtils
:   Returns a day or month number in 2 digits format.

fromArrayListToVector(ArrayList<String>) - Static method in class uk.ac.standrews.utils.main.collectionutils.CollectionUtils


fromArrayofArraysToColumnVector(String[][], int) - Static method in class uk.ac.standrews.utils.main.collectionutils.ArrayUtils
:   Converts a column of an array of arrays into a vector

fromArrayOfArraysToFile(ArrayList<ArrayList<String>>, String, String) - Static method in class uk.ac.standrews.utils.main.FileUtils
:   Writes a special-character-separated file (eg comma or tab separated) and
    fills it from an array of rows-columns.

fromArrayOfArrayToOneToManyMap(T[][]) - Static method in class uk.ac.standrews.utils.main.maputils.MapUtils
:   Array format must be [[key1, value1][key1, value2],[key2, value3],[...]].

fromArrayToArrayList(Object[]) - Static method in class uk.ac.standrews.utils.main.collectionutils.ArrayUtils
:   Creates an arrayList from an array

fromArrayToSet(String[]) - Static method in class uk.ac.standrews.utils.main.collectionutils.setutils.SetUtils


fromArrayToSet(String[]) - Static method in class uk.ac.standrews.utils.main.setutils.SetUtils


fromArrayToString(Object[]) - Static method in class uk.ac.standrews.utils.main.collectionutils.ArrayUtils
:   From an array of objects to a string.

fromArrayToString(boolean[]) - Static method in class uk.ac.standrews.utils.main.collectionutils.ArrayUtils


fromArrayToVector(Object[]) - Static method in class uk.ac.standrews.utils.main.collectionutils.ArrayUtils
:   Creates a vector from an array

fromBigSetToVector(Set<String>) - Static method in class uk.ac.standrews.utils.main.collectionutils.CollectionUtils
:   Deletes each set entry after writing it to vector, to limit memory use.

fromDMYYYtoYYYYMMDDdate(String, String, String) - Static method in class uk.ac.standrews.utils.main.TimeUtils
:   Converts from D/M/YYYY date (6/19/2006) to YYYY-MM-DD date (2006-06-19)

fromDOMtoFile(Document, String) - Static method in class uk.ac.standrews.utils.main.webutils.XMLUtils
:   From JTidy DOM to file

fromFilePathToBufferedReader(String) - Static method in class uk.ac.standrews.utils.main.FileUtils
:   From file path to bufferedReader

fromFrequencyToPercOfFrequency(TreeMap<Integer, Integer>) - Static method in class uk.ac.standrews.utils.main.stats.StatUtils
:   Converts a frequency from number of events to percentage over the total
    number of exctractions

fromInputStreamToString(InputStream) - Static method in class uk.ac.standrews.utils.main.stringutils.StringUtils


fromSetToVector(Set<String>) - Static method in class uk.ac.standrews.utils.main.collectionutils.CollectionUtils


fromStringArrayToArrayList(String[]) - Static method in class uk.ac.standrews.utils.main.collectionutils.ArrayUtils
:   Creates an arrayList from an array

fromStringToSet(String, String) - Static method in class uk.ac.standrews.utils.main.collectionutils.setutils.SetUtils


fromStringToSet(String, String) - Static method in class uk.ac.standrews.utils.main.setutils.SetUtils


fromTreeSetWithNullsToArray(TreeSet<String>) - Static method in class uk.ac.standrews.utils.main.collectionutils.setutils.SetUtils


fromTreeSetWithNullsToArray(TreeSet<String>) - Static method in class uk.ac.standrews.utils.main.setutils.SetUtils


fromTwoVectorsToMap(Vector<String>, Vector<String>) - Static method in class uk.ac.standrews.utils.main.maputils.MapUtils
:   Creates an hashmap from 2 vectors.

A B C D E F G H I J K L M N O P Q R S T U V W X Y

- Overview
- Package
- Class
- Use
- Tree
- Deprecated
- Index
- Help

- Prev Letter
- Next Letter

- Frames
- No Frames

- All Classes
